# Maternal Diet Quality during Pregnancy and Allergic and Respiratory Multimorbidity Clusters in Children from the EDEN Mother–Child Cohort

**DOI:** 10.3390/nu15010146

**Published:** 2022-12-28

**Authors:** Rosalie Delvert, Manel Ghozal, Karine Adel-Patient, Manik Kadawathagedara, Barbara Heude, Marie-Aline Charles, Isabella Annesi-Maesano, Muriel Tafflet, Bénédicte Leynaert, Raphaëlle Varraso, Blandine de Lauzon-Guillain, Annabelle Bédard

**Affiliations:** 1 Université Paris-Saclay, UVSQ, Université Paris-Sud, Inserm, Équipe d’Épidémiologie Respiratoire Intégrative, CESP, 94805 Villejuif, France; 2 Université Paris Cité, Inserm, INRAE, CRESS, 75004 Paris, France; 3 Université Paris-Saclay, CEA, INRAE, DMTS, 91191 Gif-sur-Yvette, France; 4 Unité mixte Inserm-Ined-EFS Elfe, Ined, 75020 Aubervilliers, France; 5 Desbrest Institute of Epidemiology and Public Health (IDESP), Montpellier University and Inserm, 34090 Montpellier, France

**Keywords:** maternal diet, asthma and allergic diseases, children, cluster analysis, birth cohort

## Abstract

We investigated the associations between maternal diet quality and allergic and respiratory diseases in children. Analyses were based on 1316 mother–child pairs from the EDEN mother–child cohort. Maternal diet quality during pregnancy was assessed through a food-based score (the Diet Quality), a nutrient-based score (the PANDiet), and the adherence to guidelines for main food groups. Clusters of allergic and respiratory multimorbidity clusters up to 8 years were identified using Latent Class Analysis. Associations were assessed by adjusted multinomial logistic regressions. Four clusters were identified for children: “asymptomatic” (67%, reference group), “asthma only” (14%), “allergies without asthma” (12%), “multi-allergic” (7%). These clusters were not associated with mother diet quality assessed by both scores. Children from mothers consuming legumes once a month or less were at higher risk of belonging to the “multi-allergic” cluster (odds ratio (OR) (95% confidence interval (95%CI)) = 1.60 (1.01;2.54)). No association was found with other food groups or other clusters. In our study, allergic and respiratory multimorbidity in children was described with four distinct clusters. Our results suggest an interest in legumes consumption in the prevention of allergic diseases but need to be confirmed in larger cohorts and randomized control trials.

## 1. Introduction

According to the Developmental Origins of Health and Disease (DOHaD) hypothesis, prenatal exposures, such as maternal diet during pregnancy, have long-term consequences on children’s health and development [1]. Allergic and respiratory diseases, such as asthma, most often develop in childhood and can persist into adulthood [2]. In recent decades, the prevalence of allergic and respiratory diseases has increased especially in children, affecting multiple aspects of their life [3]. Worldwide, asthma is the most common chronic disease in children with an estimated prevalence of 9.1% at 6–7 years [4] in 2015–2020. The Global Asthma Network also estimated that the prevalence in children was 7.5% for allergic rhinitis and 5.9% for eczema [4], and 6–8% of children suffer from food allergies [5]. However, although allergic and respiratory diseases are interrelated and often concomitant in children, more than what would be expected by chance alone [2], they have been considered separately in association with diet.

To date, maternal diet during pregnancy has been explored mainly through isolated nutrients or specific food groups. There is little consistent evidence on the effect of maternal diet during pregnancy on allergic and respiratory diseases, except for vitamin D and omega-3 fatty acids supplementations, associated with a reduced risk of wheezing or asthma in children [6,7,8,9]. Additionally, this approach does not consider the complexity of the diet [10].

To take this complexity into account, the mother’s overall diet has been considered through dietary patterns, either using an a priori approach with scores based on established knowledge and hypotheses or using an a posteriori exploratory approach which is data driven. A priori approaches are particularly relevant in terms of public health since they are usually based on nutritional guidelines [11,12,13,14,15]. However, studies remain sparse and findings are conflicting: two studies found no association between the Alternate Healthy Eating Index for Pregnancy (AHEI-P) and allergic and respiratory symptoms in children aged 3 and 10 years old [11,12], while a higher Healthy Eating Index (HEI-2015) during pregnancy was associated with less asthma in children aged 9 years [15]. These discordances might be explained by the heterogeneity in the scores used, the age of the children, or in the assessment of allergic and respiratory health.

We aimed to investigate the relationships between maternal diet quality during pregnancy and clusters of allergic and respiratory multimorbidity up to 8 years old in children.

## 2. Materials and Methods

### 2.1. Study Design and Population

The study is based on the EDEN mother–child cohort, a birth cohort study designed to examine early pre- and postnatal determinants of child’s development and health. Briefly, 2002 pregnant women were enrolled before 24 weeks of amenorrhea between February 2003 to January 2006 in the university hospitals of Nancy and Poitiers, France. Exclusion criteria were multiple births, known diabetes before pregnancy, French illiteracy, and planning to move out of the region in the 3 following years [16].

### 2.2. Ethics

The EDEN study was approved by the ethics research committee of Bicêtre hospital (ID 0270 of 12 December 2002) and by the National Commission on Informatics and Liberty (CNIL, ID 902267 of 12 December 2002). Upon inclusion, the mother signed a written consent for her participation and after delivery, written consent was obtained from both parents for the participation of their child.

### 2.3. Data Collection

#### 2.3.1. Maternal Diet during Pregnancy

Maternal diet during the last trimester of pregnancy was assessed through a self-administrated semi-quantitative Food Frequency Questionnaire (FFQ) including 137 items, completed after delivery and validated by 24 h recalls [17]. Frequency was evaluated with a 7-level scale from “never” to “more than once a day”. Portion size was determined using pictures on a three-level scale from the ‘SUpplementation en VItamines et Mineraux AntioXydants’ (SU.VI.MAX) portion size booklet [18] for twelve food types (meats, French fries, pastas, vegetables, cakes, cheese, etc.) or with standard portions for the French adult population. Daily food intakes were calculated by multiplying portion size (in grams) by frequency (per day). Intakes of nutrients were calculated by crossing daily food intake with the SU.VI.MAX food composition database [19].

The quality of maternal diet during pregnancy was assessed through the adherence to nutritional guidelines with two complementary scores: a score based on the guidelines for food groups intake (named in the present study the Diet Quality score) [14] and the Probability of Adequate Nutrient intake Diet quality index (PANDiet score) based on adequacy of nutrients intake [13]. The computation of the Diet Quality score is based on women’s consumption as a proportion of the guideline for each of the 17 food groups [14]. After summing the scores obtained for each item, the final score ranges from 0 to 17 points, a higher score indicates better adherence to French nutritional guidelines. The PANDiet score has been adapted and validated for French pregnant women [13] to assess adequacy of nutrient intakes according to the French nutritional references for pregnant women by using an adequation and a moderation sub-score [20]. The score ranges from 0 to 100, a higher score corresponding to a better adequacy of nutrient intake.

The intake of 11 food groups subject to recommendations in the French nutritional guidelines for women during pregnancy [21] was also calculated, in frequency or in grams according to the guideline. Food groups considered were fruit (raw or cooked), vegetables (raw or cooked), legumes, nuts, starch and grains, milk and dairy products, fish and shellfish, red meat, processed meat, poultry, and sugar-sweetened beverages (including fruit juices). Food groups with qualitative guidelines (such as “increase”/“limit”) were considered continuously: fruit (times/day), vegetables (times/day), starch and grains (times/day), poultry (g/week), and sweetened beverages (100 mL/day). Food groups with quantitative guidelines were considered as categorical variables: milk and dairy products (<3/day, ≥3/day), fish and shellfish (<2/week, ≥2/week), red meat (<500 g/week, ≥500 g/week), processed meat (<150 g/week, ≥150 g/week). Food groups with too infrequent consumption in our study population were considered as follows: legumes (≤1/month, >1/month), nuts (no consumption, consumption) (Supplementary Materials Table S1).

#### 2.3.2. Allergic and Respiratory Variables

The allergic and respiratory health of children were assessed prospectively at 8 months, 1, 2, 3, 4, 5–6 and 8 years with parental reports, using a French version of the International Study of Asthma and Allergies in Childhood (ISAAC) questionnaire [22] enriched with items on food allergies [23]. For this study, food allergy, eczema, wheezing, asthma medication, medical diagnosis of asthma between birth and 8 years and rhinitis from 1 to 8 years were used and defined as follows:

- Ever food allergy: at least one positive answer during the follow-up to the question “Has a doctor ever diagnosed your child with a food allergy?”.
- Ever eczema: at least one parental report of current eczema during the follow-up. Current eczema was characterized according to the criteria from the Mechanisms of Development of Allergy consortium (MeDALL) [2] as a positive answer to three items (“Has your child ever been diagnosed with eczema?”, “(Since last follow-up), has your child had an itchy rash (red patches, pimples, etc.) on the skin that appears and disappears intermittently?”, “Has this itchy rash affected any of the following areas: the folds of the elbows, behind the knees, in front of the ankles, under the buttocks, around the neck, around the eyes or ears?”).
- Ever wheezing: at least one positive answer during the follow-up to the question “Has your child had wheezing in the chest at any time (since last follow-up)?”.
- Ever medication for asthma attack: positive answers to two items (“(Since the last follow-up), has your child had an asthma attack?” and “Has this problem required treatment prescribed by a physician at least once?”).
- Ever asthma diagnosis: at least one positive answer during the follow-up to the question “Has your child ever been diagnosed with asthma by a doctor?”.
- Ever rhinitis: at least one parental report during the follow-up of current rhinitis. Current rhinitis was characterized according to the MeDALL criteria [2] as a positive answer to two items (“(Since last follow-up) has your child had sneezing, a runny nose or a stuffy nose without respiratory infection (no cold, no rhinopharyngitis, no flu...)?” and “Were these nose problems accompanied by watering (crying) or itching (scratching) of the eyes?”).

#### 2.3.3. Other Variables

Family characteristics were collected at inclusion or at delivery: maternal age at delivery, maternal education level (up to lower secondary, upper secondary, intermediate, 2-year university degree, ≥3-year university degree), monthly household incomes (<800EUR, 801–1500EUR, 1501–2300EUR, 2301–3000EUR, >3000EUR), primiparity, maternal smoking during pregnancy (yes/no), maternal pre-pregnancy body mass index (BMI), and family history of allergy (at least one child’s parent or sibling with food allergy, asthma, allergic rhinitis or eczema). Child’s sex, season of birth (autumn/winter, spring/summer), gestational age, and birth weight were collected in the medical file.

#### 2.3.4. Study Sample

Among 2002 women included in the cohort, 95 left the study during pregnancy or at delivery. On the 1907 newborn children, data on birth weight were available for 1899 children. Allergic and respiratory clusters were performed on 1593 children with data available for each of the six allergic and respiratory variables mentioned before.

**Figure 1:**
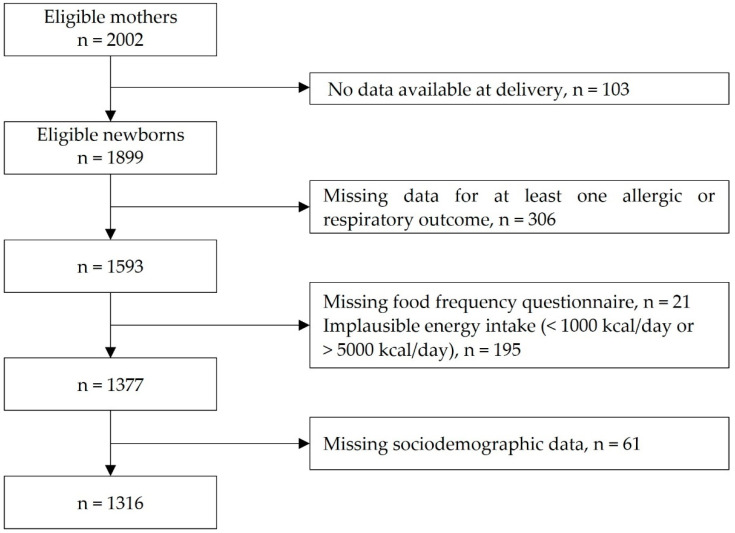
Flowchart of the study population.

We excluded mothers who did not complete the FFQ (*n* = 21), and those with implausible energy intake (<1000 kcal/day or >5000 kcal/day, *n* = 195). On the 1377 remaining mother–child pairs, 1316 had complete data for maternal and sociodemographic characteristics and were involved in the main analyses (Figure 1).

### 2.4. Statistical Analyses

To investigate a potential attrition bias, included mother–child pairs (*n* = 1316) were compared to excluded pairs (*n* = 646) regarding sociodemographic characteristics collected at inclusion with Student *t*-test for continuous variables and Pearson chi-square test for categorical variables.

Unsupervised allergic and respiratory clusters were constructed using Latent Class Analysis (LCA) with the aim of identifying groups of children with similar allergic and respiratory profiles up to 8 years. LCA was conducted on the 1593 children with data available for the 6 synthetic variables mentioned before (ever food allergy, eczema, wheezing, asthma medication, medical diagnosis of asthma, and rhinitis). Solutions of 2 to 6 clusters were tested using 100 replications and 100,000 iterations. Bayesian Information Criterion (BIC) minimization and interpretability were used to select the optimal number of clusters. Allergic and respiratory multimorbidity clusters were described according to the variables used for cluster construction (summary variables on the 0-to-8-year period), to allergic and respiratory outcomes at each time, and to sociodemographic variables not included in the cluster construction.

Associations between dietary variables and allergic and respiratory clusters were assessed with multinomial logistic regressions on the complete-case sample. Analyses were run separately for each dietary variable (each score and each food group). Unadjusted analyses were performed using Pearson chi-square tests and ANOVA tests, according to the type of dietary exposure. For adjusted analyses, the following potential confounders were identified from the literature and selected using the Directed Acyclic Graph (DAG) method (Supplementary Materials Figure S1): maternal characteristics (age at delivery, education, monthly household income, smoking status during pregnancy, pre-pregnancy BMI, primiparity), family history of allergy, and season of birth. All models were also adjusted for total energy intake, child’s sex, and recruitment center (Nancy, Poitiers) and analyses by food groups were further adjusted for the PANDiet score to account for the global quality of the diet.

Interactions between maternal diet and (1) family history of allergy, (2) child’s sex, and (3) maternal smoking during pregnancy were tested. As no significant interaction was found, analyses were not stratified.

For all analyses, associations with *p*-values < 0.05 were considered statistically significant.

LCA were performed using R software version 4.1.2 (R Foundation for Statistical Computing, Vienna, Austria) and all other analyses were performed using V9.4 SAS (SAS Institute Inc., Cary, NC, USA).

## 3. Results

### 3.1. Sample Characteristics

General maternal and perinatal characteristics for the overall included population are described in Table 1. Among the 1316 mothers included, mean age at delivery was 29.7 years and 52.6% of the included children were male. Some differences were found between included mothers and excluded ones (Supplementary Materials Table S2). Compared with excluded mothers, included mothers were older, had a higher socio-economic status, were more frequently born in France, had less frequently smoked during pregnancy, had a lower pre-pregnancy BMI, and were more frequently primiparous. Included children had more frequently allergic parents or siblings and were more frequently born in spring or summer compared to excluded children. No difference was found for recruitment centre and child’s sex.

### 3.2. Identification of Allergic and Respiratory Clusters

Using LCA on 1593 children, the 4-class solution was found to have lowest BIC (BIC = 8550.7) and good interpretability. These four clusters were identified according to their allergic and respiratory characteristics (Table 2, Supplementary Materials Table S3) as follows:

- “asymptomatic” cluster, (67%): This cluster corresponded to children with low prevalence of all allergic and respiratory outcomes considered in the LCA, in comparison with other clusters.
- “asthma only” cluster, (14%): A cluster characterised by a high prevalence of respiratory outcomes—wheezing (94.6%), asthma medication (90.1%), and medical diagnosis of asthma (76.7%)—and a low prevalence of food allergies (4.9%) and eczema (7.2%).
- “allergies without asthma” cluster, (12%): in this cluster children had a high prevalence of food allergy (40.2%), eczema (94.0%), and rhinitis (50.0%), but a low prevalence of asthma medication (1.1%), and medical diagnosis of asthma (6.5%).
- “multi-allergic” cluster, (7%): the prevalence of all allergic and respiratory outcomes considered in the LCA was high.

Sociodemographic and perinatal description of the clusters is given in Supplementary Materials Table S4.

### 3.3. Maternal Diet Quality and Allergic and Respiratory Multimorbidity Clusters

Associations between maternal diet quality and allergic and respiratory multimorbidity clusters were performed considering the “asymptomatic” cluster as the reference group for all analyses. In both unadjusted (Table 3) and adjusted analyses (Table 4), no association was found between the Diet Quality score nor the PANDiet score with allergic and respiratory multimorbidity clusters.

Considering food groups, maternal consumption of legumes once a month or less was associated with a higher risk for the child to belong to the “multi-allergic” cluster compared with the “asymptomatic” cluster (odds ratio (OR) (95% confidence interval (95%CI) = 1.60 (1.01;2.54)). The same trend was observed for the “asthma only” cluster, although not reaching significance (1.37 (0.98;1.91), *p* = 0.06). No association was found for other food groups or clusters.

## 4. Discussion

Using an unsupervised clustering method, allergic and respiratory multimorbidity in children up to 8 years was described by defining four distinct clusters: “asymptomatic”, “asthma only”, “allergies without asthma”, and “multi-allergic”. Although the quality of pregnant women’s diet assessed by global scores was not associated with these allergic and respiratory multimorbidity clusters in our study, no or infrequent consumption of legumes during pregnancy was associated with a higher risk of belonging to the “multi-allergic” cluster in children. No association was found for fruit, vegetables, starch and grains, nuts, milk and dairy products, fish and shellfish, red meat, poultry, processed meat, and sugar-sweetened beverages.

Over the last years, new methods were used to identify allergic phenotypes with data-driven approaches. Studies mainly focused on one specific allergic or respiratory disease and the concept of multimorbidity has been poorly studied [2]. However, identifying clusters provides new information on the inter-relations between allergic and respiratory diseases in children. A pooled population of European birth cohorts at 4 and 8 years showed with unsupervised methods that children suffering from asthma, rhinitis, or eczema are better classified together than separately [24]. A recent Australian study constructed allergic and respiratory clusters on children aged 12–13 years and selected four clusters similar to ours [25].

To our knowledge, maternal diet quality during pregnancy has never been studied in association with allergic and respiratory diseases considered as multimorbidity clusters. Some studies reported no association between healthy eating, assessed by AHEI-P [11,12] or a principal component analysis (PCA) approach [26,27], and wheezing [11,26,27], asthma [11,27], eczema [11,26,27], or atopy [12,27]. However, other studies showed an increased risk of eczema at 1 [28] and 7–9 years [29] related to diet quality assessed by PCA and a priori score, respectively. A prospective longitudinal study showed an association between a higher HEI-2015 score during pregnancy and a lower prevalence of asthma in children over a 10-year follow-up [15]. Therefore, the current level of evidence is not sufficient to conclude that maternal diet quality during pregnancy is associated with allergic and respiratory diseases. Heterogeneity in the age of children (1 year to over 10 years), the allergic outcomes, and the methods used to assess diet quality may explain part of the inconsistency of the results.

Our results highlighted a weak relation between infrequent legumes consumption during pregnancy and a higher risk of combining multiple allergies and suggested a higher risk of asthma without allergic diseases in children. To our knowledge, legumes consumption has been poorly studied in the literature, but previous studies reported that legumes and nuts intakes were not associated with the risk of inhalant or food allergens at 5 years [30] or with respiratory symptoms in infants [15]. Similarly, no association was found between the consumption of beans or bean products and the risk of food allergy or eczema at 1 year [31], but consumption of natto, a traditional Japanese fermented soybean food, was associated with a lower risk of eczema at 6 months [32]. Finally, a study investigating the role of legumes consumption on allergic diseases found a protective association with persistent wheeze at 6.5 years that almost reached significance [33]. A potential explanation for our results may be that the high fiber content of legumes favors a healthy maternal gut microbiota that may be beneficial for the development of the child’s immune system [34]. However, this association was not found in other fiber-rich foods, such as fruit or vegetables. The association was highlighted only for the multi-allergic cluster, as children from this group may represent more severe cases. Unfortunately, legumes consumption was too infrequent in our population to investigate the potential effect of adherence to the French nutritional guidelines of at least twice a week [21], studies in larger cohorts are needed to further explore this hypothesis.

Fruit and vegetables consumption during pregnancy has been largely studied in the literature and, consistent with our results, most studies reported no association between fruit [33,35,36,37,38,39,40,41] or vegetables [30,35,38,39,40,41,42,43,44] consumption during pregnancy and allergic or respiratory outcomes in children. However, a previous study conducted at 3 years in the same cohort [37] found an association between raw and cooked green vegetables consumption during pregnancy and a lower risk of allergic rhinitis in children. These differences may be explained by the age difference and by the fact that allergic and respiratory outcomes in the previous study were considered separately and not as multimorbidity clusters.

Studies investigating the role of starches and cereals on allergic and respiratory diseases have led to mixed findings. Five studies did not report association between starch or grains consumption during pregnancy and the risk of wheezing [35,37], asthma [35,37], rhinitis [35,37,40], eczema [35,37,40], or food allergy [30,43]. However, it was associated with a reduced risk of wheezing in two studies [33,40] and with a lower risk of atopic dermatitis in one study [45]. This heterogeneity may be explained by the hypothesis on fibers mentioned before: wholegrain cereals would be more likely to have a protective effect due to their high fiber content. However, in our population, the consumption of wholegrain products was too infrequent to investigate a potential specific effect of wholegrain cereals. 

Fish and shellfish consumption during pregnancy was extensively studied, leading to heterogeneous results. Some studies showed no association between fish consumption during pregnancy and allergic outcomes in children, as in our study [32,37,39,43,46,47,48,49]; other studies reported that fish intake was associated with either a lower risk of eczema [35,50,51] or a higher risk of eczema [52] and either a lower risk of food allergy [53] or a high risk of food allergy [31]. One hypothesis to explain these disparities may be that fish, especially oily fish, provides long-chain polyunsaturated fatty acids (PUFAs) with potential beneficial effects on allergy that may be counterbalanced by food chemicals in fish [54,55]. Investigations in larger cohorts and in populations with more diverse fish intakes may help to investigate this hypothesis.

In this longitudinal study, the population included in the EDEN cohort had a higher socioeconomic level than the general French population, which was reinforced by attrition bias [16]. It would be interesting to replicate such analyses in other samples including more vulnerable populations, as a higher socioeconomic level can be associated with a better adherence to dietary guidelines [56], which may partly explain the absence of association found with diet quality in this study. A strength of our study is that data on allergic and respiratory outcomes and dietary exposures were collected through validated questionnaires [17,22]. Moreover, allergic and respiratory diseases were considered jointly through an unsupervised clustering method to take into account their interrelations. It was possible to have a comprehensive view of allergic and respiratory multimorbidity up to 8 years thanks to repeated data on the main allergic and respiratory symptoms affecting children, including detailed validated information on respiratory symptoms. However, our clusters are based on the ever diagnosis or parental report of allergic and respiratory diseases, hence classification bias cannot be excluded. In addition, our analyses cannot provide information on the allergic march of individuals that could be explored with longitudinal approaches. Finally, the number of mother–child pairs included in the final analyses was limited and the dispersion of dietary scores was low, thus replication in larger cohorts with more diverse dietary intakes would help to confirm our results.

## 5. Conclusions

In conclusion, we found four different clusters to describe allergic and respiratory diseases up to 8 years in children. Maternal diet quality during pregnancy was not associated with allergic and respiratory multimorbidity clusters up to 8 years. However, infrequent legumes consumption was associated with a higher risk of suffering from multiple allergies and respiratory diseases (food allergy, eczema, wheezing, asthma, and rhinitis) during childhood. There are a lot of inconsistencies in the literature concerning these associations, but as our study is the first accounting for allergic and respiratory multimorbidity in association with maternal diet, further research is needed to confirm these results.

## Figures and Tables

**Table 1:** Characteristics of the study population (*n* = 1316).

	% (*n*) or Mean ± sd
Center	
Poitiers	49.2% (647)
Nancy	50.8% (669)
Maternal age at delivery (years)	29.7 ± 4.8
Maternal education level	
Up to lower secondary	4.2% (55)
Upper secondary	18.4% (242)
Intermediate	17.9% (235)
2-year university degree	23.8% (313)
≥3-year university degree	35.8% (471)
Household income (euros/month)	
<800	2.9% (38)
801–1500	9.9% (130)
1501–2300	28.1% (370)
2301–3000	28.8% (379)
>3000	30.3% (399)
Smoking during pregnancy	22.9% (301)
Pre-pregnancy BMI (kg/m^2^)	23.1 ± 4.3
Primiparity	46.2% (608)
Season of birth	
Autumn/winter	45.5% (599)
Spring/summer	54.5% (717)
Boys	52.6% (692)
Family history of allergy	53.0% (698)
Gestational age (weeks)	39.3 ± 1.6
Birth weight (g)	3290 ± 494

BMI: Body Mass Index; sd: standard deviation.

**Table 2:** Prevalence of allergic and respiratory diseases in children up to 8 years according to the allergic and respiratory multimorbidity clusters (*n* = 1593).

	Total	Allergic and Respiratory Multimorbidity Clusters			
		Asymptomatic	Asthma Only	Allergies without Asthma	Multi-Allergic
	*n* = 1593	*n* = 1075	*n* = 223	*n* = 184	*n* = 111
Food allergy (0–8 years)	11.4% (181)	4.8% (52)	4.9% (11)	40.2% (74)	39.6% (44)
Eczema (0–8 years)	26.0% (414)	11.2% (120)	7.2% (16)	94.0% (173)	94.6% (105)
Wheezing (0–8 years)	40.4% (643)	21.4% (230)	94.6% (211)	51.1% (94)	97.3% (108)
Asthma medication (0–8 years)	21.0% (335)	2.0% (21)	90.1% (201)	1.1% (2)	100.0% (111)
Asthma diagnosis (0–8 years)	18.5% (295)	0.7% (8)	76.7% (171)	6.5% (12)	93.7% (104)
Rhinitis (1–8 years)	20.0% (319)	10.8% (116)	19.7% (44)	50.0% (92)	60.4% (67)

Values are the prevalence of each variable included in the clusters construction in % (*n*).

**Table 3:** Maternal diet characteristics by allergic and respiratory multimorbidity cluster up to 8 years in children (*n* = 1316).

	Total	Allergic and Respiratory Multimorbidity Clusters				
		Asymptomatic	Asthma Only	Allergies without Asthma	Multi-allergic	*p*-Value ^†^
	***n* = 1316**	***n* = 880**	***n* = 190**	***n* = 154**	***n* = 92**	
Diet Quality score (range 0–17)	12.1 ± 1.2	12.1 ± 1.2	12.0 ± 1.2	12.1 ± 1.2	12.0 ± 1.2	0.74
PANDiet score (range 0–100)	64.3 ± 6.9	64.3 ± 6.9	64.3 ± 7.1	64.6 ± 7.0	64.3 ± 6.7	0.95
Fruit (times/day)	1.5 ± 1.6	1.5 ± 1.5	1.7 ± 2.0	1.4 ± 1.3	1.6 ± 1.8	0.55
Vegetables (times/day)	1.8 ± 1.2	1.7 ± 1.2	1.9 ± 1.2	1.8 ± 1.1	1.8 ± 1.2	0.78
Legumes (>1/month)	47.1% (620)	48.1% (423)	43.2% (82)	50.0% (77)	41.3% (38)	0.35
Starch and grains (times/day)	2.8 ± 1.1	2.8 ± 1.2	2.8 ± 1.2	2.7 ± 1.2	2.8 ± 0.9	0.82
Nuts (consumption)	49.5% (652)	49.1% (432)	50.0% (95)	48.7% (75)	54.3% (50)	0.81
Milk and dairy products (≥3 times/day)	72.3% (952)	72.4% (637)	73.7% (140)	69.5% (107)	73.9% (68)	0.82
Fish and shellfish (≥2 times/week)	26.3% (346)	25.9% (228)	31.6% (60)	24.7% (38)	21.7% (20)	0.26
Red meat (<500 g/week)	79.5% (1046)	79.4% (699)	81.1% (154)	77.3% (119)	80.4% (74)	0.85
Processed meat (<150 g/week)	73.3% (965)	72.7% (640)	73.2% (139)	76.6% (118)	73.9% (68)	0.79
Poultry (g/week)	136 ± 131	135 ± 132	142 ± 134	137 ± 131	138 ± 107	0.97
Sugar-sweetened beverages (mL/day)	294 ± 400	287 ± 398	341 ± 438	235 ± 306	346 ± 462	0.03 *
Total energy intake (kcal/J)	2191 ± 729	2161 ± 707	2297 ± 812	2183 ± 735	2272 ± 734	0.08

Values are % (*n*) or mean ± standard deviation. ^†^ *p*-values of Pearson chi-square test for categorical dietary exposures and of ANOVA test for continuous dietary exposures. * *p*-value < 0.05.

**Table 4:** Adjusted association between maternal diet during pregnancy and allergic and respiratory multimorbidity clusters up to 8 years of age (*n* = 1316).

	Allergic and Respiratory Multimorbidity Clusters (Ref = Asymptomatic)		
	**Asthma Only**	**Allergies without Asthma**	**Multi-Allergic**
Diet Quality score (range 0–17)	1.01 (0.86;1.18)	1.05 (0.89;1.24)	1.01 (0.82;1.25)
PANDiet score (range 0–100)	0.91 (0.71;1.17)	1.09 (0.83;1.42)	0.91 (0.65;1.28)
Fruit (times/day)	1.05 (0.95;1.17)	0.93 (0.81;1.07)	1.03 (0.89;1.20)
Vegetables (times/day)	1.12 (0.97;1.30)	1.00 (0.84;1.19)	1.07 (0.87;1.32)
Legumes			
≤1/month	1.37 (0.98;1.91)	0.98 (0.69;1.40)	1.60 (1.01;2.54) *
>1/month	1 (Ref)	1 (Ref)	1 (Ref)
Starch and grains (times/day)	0.98 (0.84;1.15)	0.91 (0.76;1.08)	1.05 (0.84;1.30)
Nuts			
No consumption	1.05 (0.75;1.47)	1.02 (0.71;1.46)	0.90 (0.57;1.43)
Consumption	1 (Ref)	1 (Ref)	1 (Ref)
Milk and dairy products			
<3 times/day	1.01 (0.67;1.53)	1.30 (0.85;2.00)	0.98 (0.56;1.71)
≥3 times/day	1 (Ref)	1 (Ref)	1 (Ref)
Fish and shellfish			
<2 times/week	0.75 (0.51;1.10)	1.12 (0.73;1.74)	1.35 (0.76;2.39)
≥2 times/week	1 (Ref)	1 (Ref)	1 (Ref)
Red meat			
<500 g/week	1 (Ref)	1 (Ref)	1 (Ref)
≥500 g/week	0.78 (0.51;1.19)	1.10 (0.71;1.70)	0.83 (0.46;1.49)
Processed meat			
<150 g/week	1 (Ref)	1 (Ref)	1 (Ref)
≥150 g/week	0.76 (0.52;1.13)	0.79 (0.51;1.21)	0.74 (0.43;1.25)
Poultry (g/week)	1.01 (0.95;1.07)	1.00 (0.94;1.08)	1.00 (0.92;1.09)
Sugar-sweetened beverages (mL/day)	1.00 (0.96;1.04)	0.96 (0.91;1.02)	1.01 (0.95;1.07)

Adjusted OR (95%CI) from multinomial logistic regressions. Each dietary exposure was considered in a separate model. Models for diet quality scores are adjusted for maternal characteristics (age at delivery, education level, household income, smoking status during pregnancy, pre-pregnancy body mass index (BMI), primiparity, and total energy intake), child’s sex, family history of allergy, season of birth, and center. For food groups, models were also adjusted on the PANDiet score. * *p*-value < 0.05. OR, odds ratio; CI, confidence interval.

## Data Availability

The data underlying the findings cannot be made freely available for ethical and legal restrictions imposed because this study includes a substantial number of variables that, together, could be used to re-identify the participants based on a few key characteristics and then be used to have access to other personal data. Therefore, the French ethics authority strictly forbids making these data freely available. However, they can be obtained upon request from the EDEN principal investigator. Readers may contact barbara.heude@inserm.fr to request the data. The analytic code will be made available upon request pending application and approval.

## Supplementary Materials

The following supporting information can be downloaded at: https://www.mdpi.com/article/10.3390/nu15010146/s1, Figure S1: Directed Acyclic Graph (DAG) on maternal diet during pregnancy and allergic and respiratory diseases in children, used for the selection of cofounders; Table S1: Composition of food groups used in the data analyses; Table S2: Comparison of included and excluded population on inclusion parameters; Table S3: Detailed allergic and respiratory characteristics of allergic and respiratory multimorbidity clusters up to 8 years (*n* = 1593); Table S4: Sociodemographic and perinatal characteristics of allergic and respiratory multimorbidity clusters up to 8 years (*n* = 1593).
