# Supplementary material for: Maternal Diet Quality during Pregnancy and Allergic and Respiratory Multimorbidity Clusters in Children from the EDEN Mother–Child Cohort
Source: Nutrients. 2022 Dec 28;15(1):146. doi: 10.3390/nu15010146 (PMC9824220; doi:10.3390/nu15010146)
Supplement: Supplementary file 1 [file nutrients-15-00146-s001.zip › nutrients-2079184-supplementary.pdf]

Table S1. Composition of food groups used in the data analyses.

Food groups	Food components
Fruit	Apricots, melon, mangoes; peaches, prunes, cherries; strawberries, raspberries; bananas; kiwi; citrus fruit (oranges, mandarins, grapefruit); apples or pears; grapes; other fresh fruit (pineapple); dried apricots or peaches; other dried fruit
Vegetables	Green beans; raw and cooked endives, spinach, cress; leek, cabbage (green, cauliflower, Brussels sprouts); broccoli; cooked carrots; zucchini, eggplant (ratatouille); peas; other vegetables (turnip, chard); vegetable soup; corn; pumpkin, sweet potatoes; green salad; grated carrot; other raw vegetables (celery, tomatoes, beets, cabbage, cucumber, radish); avocado
Legumes	Lentils, white beans, chickpeas, broad beans
Starch and grains	Mashed potatoes; boiled or baked potatoes; fried potatoes; bread (white bread or sandwich bread); wholegrain or special bread; rusks or crackers or toast; breakfast cereals (corn flakes, cheerios, chocolate, puffed cereals, muesli); pasta (macaroni, spaghetti, coquillettes); ravioli and filled pasta; rice, semolina or wheat; potato gratin
Nuts	Nuts, hazelnuts, almond; peanuts
Milk and dairy products	Emmental, Gruyère, Comté, Beaufort; Bonbel, Babybel, Gouda, Edam, Cantal, Tommes, Morbier, St. Nectaire, Reblochon; Brie, Camembert, Pont l'Evêque, Munster, Vacherin, St Marcelin, Caprice des Dieux-type cheeses; Roquefort, blue cheeses; goat cheese; fresh cheese (Tartare, Kiri); whole milk; semi-skimmed milk; skimmed milk; cream; light cream; whipped cream; ice cream; cottage cheese or 0% fat yoghurt (plain, with fruit); cottage cheese with 20%, 30% or 40% fat; yoghurts (plain, fruit, flavoured); desserts (cream desserts, mousses, flans)
Fish and shellfish	Fresh or frozen fish (cod, pollack, whiting, sole, trout); canned fish in oil (tuna, sardines); smoked or salted fish (salmon, herring); breaded fish; fish-based ready-made dishes; shellfish (mussels, oysters, shrimp)
Red meat	Liver (heifer, poultry); tongue, tripe, blood sausage, andouillettes, sweetbreads, kidneys; beef (excluding ground steak); ground beef steak; pork (excluding processed meat); veal meat; lamb, mutton meat
Processed meat	Dry sausage (or salami); mortadella, saveloy; pâté, rillettes; ham (white, cures, bacon); sausage (chipolata sausages, merguez, Strasbourg sausage)
Poultry	Poultry (chicken, turkey), rabbit
Sugar-sweetened beverages	Orange juice, grapefruit juice, pineapple juice, apple juice, grape juice; syrup; non-light cola; lemonade, sugar-sweetened sodas

Foods separated with a "," are from the same item, foods separated with a ";" are from different item.

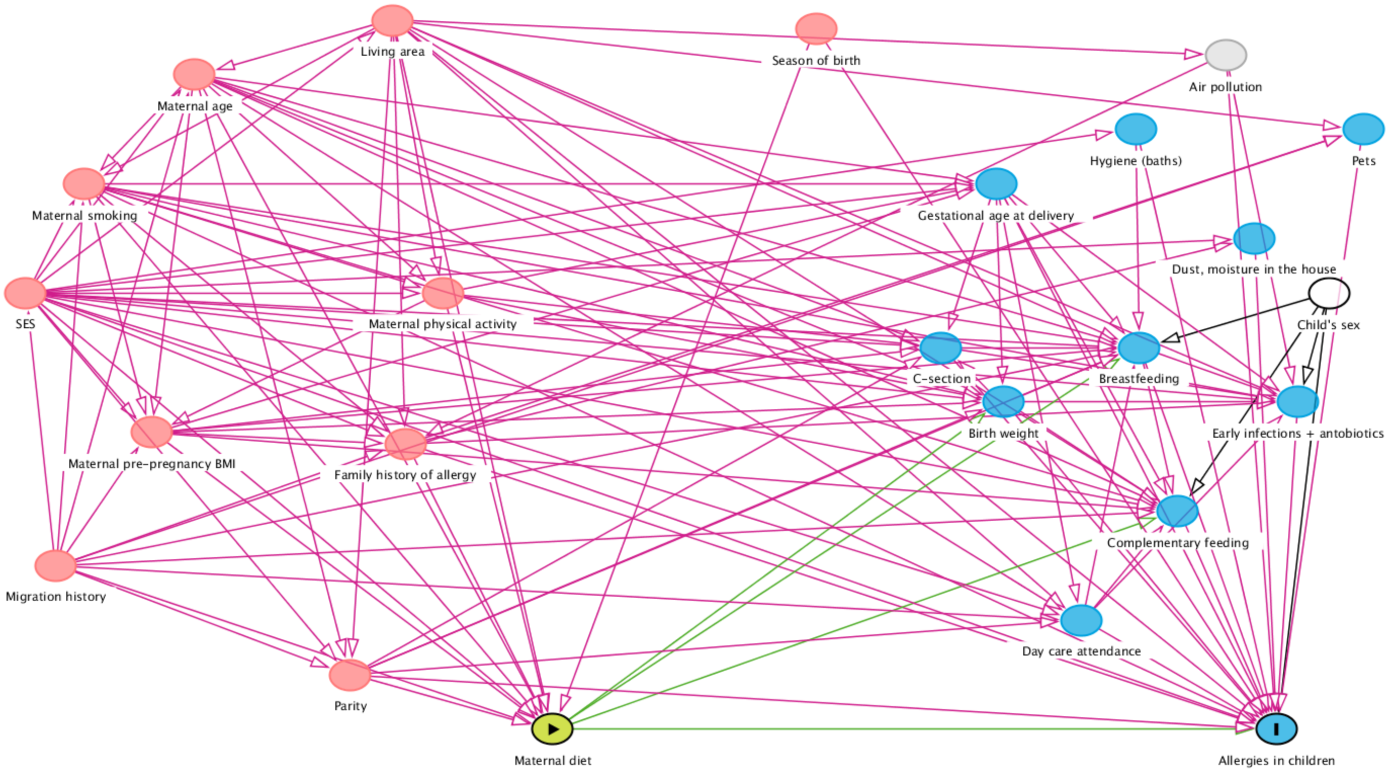

Figure S1. Directed Acyclic Graph (DAG) on maternal diet during pregnancy and allergic and respiratory diseases in children, used for the selection of cofounders. This graph was made with DAGitty v3.1. (www.dagitty.net, accessed 15 July 2022). The triangle in a green circle represents the main exposure (maternal diet during pregnancy) and the I in a blue circle is the outcome (allergies in children). Red circles represent potential cofounders (i.e., ancestors of exposure and outcome), intermediate factors (i.e., ancestor of the outcome but not of the exposure) are represented with blue circles. Unobservable (latent) variables are in a grey circle and variables included in the adjustment that are not cofounders are represented in a white circle. Arrows indicate the direction of causal paths. SES: socio-economic status.

Table S2. Comparison of included and excluded population on inclusion parameters.

	**Excluded population** ***n*** **= 686**	**Included population** ***n*** **= 1316**	***p*-value †**
Center			0.31
Poitiers	46.8% (321)	49.2% (647)	
Nancy	53.2% (365)	50.8% (669)	
Maternal age at delivery (years)	29.0 ± 5.1	29.7 ± 4.8	0.002 *
Maternal education level			<0.0001 *
Up to lower secondary	15.0% (89)	4.2% (55)	
Upper secondary	27.4% (163)	18.4% (242)	
Intermediate	17.7% (105)	17.9% (235)	
2-year university degree	17.0% (101)	23.8% (313)	
≥ 3-year university degree	22.9% (136)	35.8% (471)	
Household income (euros/month)			<0.0001 *
< 800	9.2% (55)	2.9% (38)	
801–1500	17.4% (104)	9.9% (130)	
1501–2300	33.2% (198)	28.1% (370)	
2301–3000	20.4% (122)	28.8% (379)	
> 3000	19.8% (118)	30.3% (399)	
Mother born abroad	4.2% (25)	0.8% (11)	<0.0001 *
Smoking during pregnancy	34.5% (183)	22.9% (301)	<0.0001 *
Pre-pregnancy BMI (kg/m^2)	23.6 ± 5.4	23.1 ± 4.3	0.04 *
Season of birth			0.0002 *
Autumn/winter	54.4% (373)	45.5% (599)	
Spring/summer	45.6% (313)	54.5% (717)	
Primiparity	40.9% (240)	46.2% (608)	0.03*
Boys	52.5% (308)	52.6% (692)	0.96
Family history of allergy	42.3% (290)	53.0% (698)	<0.0001*

Values are % (*n*) or mean ± standard deviation. † *p*-values of Pearson chi-square test for categorical variables and of Student t-test for continuous variables. * *p*-value < 0.05. BMI: Body Mass Index.

Table S3. Detailed allergic and respiratory characteristics of allergic and respiratory multimorbidity clusters up to 8 years (n = 1593).

	**Total**	**Allergic and respiratory multimorbidity clusters**			
		asymptomatic	asthma only	allergies without asthma	multi-allergic
	n = 1593	n = 1075	n = 223	n = 184	n = 111
Food allergy reported at					
1 year †	4.4% (67)	1.2% (12)	1.4% (3)	17.9% (32)	18.0% (20)
2 years †	4.7% (67)	1.1% (10)	2.1% (4)	19.3% (33)	20.6% (20)
3 years †	5.2% (68)	1.8% (15)	1.6% (3)	15.4% (25)	25.3% (25)
4 years †	5.3% (65)	2.0% (16)	2.9% (5)	14.7% (22)	23.9% (22)
5 years †	6.6% (78)	2.3% (18)	2.4% (4)	19.6% (30)	28.3% (26)
8 years †	6.7% (59)	2.7% (16)	3.4% (4)	19.5% (22)	27.4% (17)
Eczema reported at					
1 year	14.6% (229)	5.3% (56)	4.5% (10)	55.7% (102)	55.0% (61)
2 years	9.7% (137)	4.1% (39)	3.1% (6)	34.5% (59)	32.7% (33)
3 years	11.2% (146)	4.3% (37)	2.2% (4)	40.2% (66)	39.8% (39)
4 years	11.1% (136)	4.0% (32)	1.7% (3)	39.7% (60)	44.1% (41)
5 years	9.4% (112)	4.0% (31)	1.8% (3)	31.4% (48)	32.6% (30)
8 years	9.8% (86)	4.3% (25)	0.0% (0)	30.1% (34)	43.5% (27)
Wheezing reported at					
1 year	24.1% (376)	12.5% (131)	59.5% (132)	27.5% (50)	56.8% (63)
2 years	16.2% (231)	7.8% (75)	46.1% (88)	16.5% (28)	39.2% (40)
3 years	12.9% (168)	3.6% (31)	37.2% (68)	15.3% (25)	45.4% (44)
4 years	13.1% (159)	4.0% (32)	35.5% (61)	10.0% (15)	54.8% (51)
5 years	12.4% (147)	3.5% (27)	39.2% (65)	9.2% (14)	45.6% (41)
8 years	8.6% (75)	1.5% (9)	23.1% (27)	6.3% (7)	51.6% (32)
Asthma medication reported at					
1 year	8.6% (134)	0.2% (2)	38.3% (85)	0.0% (0)	42.3% (47)
2 years	5.0% (70)	0.1% (1)	23.4% (44)	0.0% (0)	25.5% (25)
3 years	6.5% (83)	0.0% (0)	25.6% (46)	0.0% (0)	39.8% (37)
4 years	8.4% (102)	0.5% (4)	30.6% (52)	0.0% (0)	50.5% (46)
5 years	11.3% (141)	1.8% (15)	40.2% (72)	0.6% (1)	54.6% (53)
8 years	6.7% (59)	0.2% (1)	20.7% (24)	0.9% (1)	53.2% (33)
Asthma diagnosis reported at					
1 year †	8.8% (137)	0.1% (1)	38.7% (86)	1.6% (3)	42.3% (47)
2 years †	5.2% (73)	0.2% (2)	23.0% (43)	0.0% (0)	28.9% (28)
3 years †	7.4% (95)	0.1% (1)	27.9% (50)	1.9% (3)	44.1% (41)
4 years †	9.1% (110)	0.3% (2)	36.5% (61)	0.0% (0)	51.6% (47)
5 years †	11.6% (138)	0.5% (4)	43.7% (73)	3.9% (6)	60.4% (55)
8 years †	10.0% (88)	0.2% (1)	38.8% (45)	2.7% (3)	62.9% (39)
Rhinitis reported at					
2 years	7.4% (105)	4.2% (40)	6.9% (13)	20.0% (34)	18.2% (18)
3 years	8.6% (113)	3.7% (32)	6.5% (12)	21.2% (35)	34.7% (34)
4 years	7.3% (89)	2.8% (22)	7.0% (12)	12.7% (19)	40.0% (36)
5 years	8.6% (102)	4.0% (31)	9.1% (15)	17.2% (26)	33.3% (30)
8 years	9.6% (84)	4.1% (24)	12.0% (14)	18.6% (21)	40.3% (25)

Values are % (n), †: based on a diagnosis by a physician since birth. Food allergy was defined as the positive answer to the question “Has a doctor ever diagnosed your child with a food allergy?”. Eczema was defined as a positive answer to three items: “Has your child ever been diagnosed with eczema?”, “(Since last follow-up), has your child had an itchy rash (red patches, pimples, etc.) on

the skin that appears and disappears intermittently?", "Has this itchy rash affected any of the following areas: the folds of the elbows, behind the knees, in front of the ankles, under the buttocks, around the neck, around the around the eyes or ears?". Wheezing was defined by the positive answer to the question "Has your child had wheezing in the chest at any time (since last follow-up)?". Medication for asthma was defined as the positive answers to two items: "(Since the last follow-up), has your child had an asthma attack?" and "Has this problem required at least one treatment prescribed by a physician?". Asthma diagnosis was defined as the positive answer to the question "Has your child ever been diagnosed with asthma by a doctor?" Rhinitis was defined as a positive answer to two items: "(Since last follow-up) has your child had sneezing, a runny nose or a stuffy nose without respiratory infection (no cold, no rhinopharyngitis, no flu...)? Were these nose problems accompanied by watering (crying) or itching (scratching) of the eyes?". The allergic and respiratory variables presented in this table were not used in the construction of the clusters.

Table S4. Sociodemographic and perinatal characteristics of allergic and respiratory multimorbidity clusters up to 8 years (n = 1593).

	**Allergic and respiratory multimorbidity clusters**			
	asymptomatic	asthma only	allergies without asthma	multi-allergic
	n = 1075	n = 223	n = 184	n = 111
Center				
Poitiers	48.2% (518)	57.8% (129)	40.8% (75)	63.1% (70)
Nancy	51.8% (557)	42.2% (94)	59.2% (109)	36.9% (41)
Maternal age at delivery (years)	29.8 ± 4.8	29.6 ± 4.8	30.0 ± 4.8	29.3 ± 4.5
Maternal education				
Up to lower secondary	5.2% (56)	4.1% (9)	6.0% (11)	4.5% (5)
Upper secondary	18.4% (196)	24.8% (55)	20.7% (38)	20.7% (23)
Intermediate	18.1% (193)	22.5% (50)	10.9% (20)	20.7% (23)
2-year university degree	22.4% (239)	20.3% (45)	23.9% (44)	25.2% (28)
≥ 3-year university degree	36.0% (384)	28.4% (63)	38.6% (71)	28.8% (32)
Household income (euros /month)				
< 800	3.8% (41)	1.8% (4)	2.2% (4)	3.6% (4)
801–1500	9.7% (104)	9.0% (20)	10.9% (20)	13.5% (15)
1501–2300	28.6% (305)	35.1% (78)	25.0% (46)	33.3% (37)
2301–3000	26.7% (285)	27.9% (62)	32.6% (60)	30.6% (34)
> 3000	31.1% (332)	26.1% (58)	29.3% (54)	18.9% (21)
Smoking during pregnancy	22.1% (232)	21.5% (47)	29.4% (53)	27.5% (30)
Pre-pregnancy BMI (kg/m^2)	23.0 ± 4.5	24.0 ± 4.9	22.8 ± 3.6	23.2 ± 4.3
Primiparity	47.3% (507)	43.2% (96)	44.0% (81)	45.0% (50)
Season of birth				
Autumn/winter	44.9% (483)	44.4% (99)	50.5% (93)	45.9% (51)
Spring/summer	55.1% (592)	55.6% (124)	49.5% (91)	54.1% (60)
Boys	49.0% (527)	64.6% (144)	48.9% (90)	60.4% (67)
Family history of allergy	46.3% (498)	61.0% (136)	65.8% (121)	75.7% (84)
Gestational age (weeks)	39.4 ± 1.5	39.1 ± 2.1	39.2 ± 1.6	38.8 ± 2.3
Birth weight (g)	3291 ± 472	3287 ± 562	3303 ± 568	3183 ± 569

Values are % (n) or mean ± standard deviation; BMI: Body Mass Index.
